# Associations between Cord Blood Leptin Levels and Childhood Adiposity Differ by Sex and Age at Adiposity Assessment

**DOI:** 10.3390/life12122060

**Published:** 2022-12-08

**Authors:** Kasandra Blais, Myriam Doyon, Mélina Arguin, Luigi Bouchard, Patrice Perron, Marie-France Hivert

**Affiliations:** 1 Department of Medicine, Faculty of Medicine and Health Sciences, Université de Sherbrooke, Sherbrooke, QC J1H 5N4, Canada; 2 Centre de Recherche du Centre Hospitalier Universitaire de Sherbrooke, Sherbrooke, QC J1H 5N4, Canada; 3 Department of Biochemistry and Functional Genomics, Faculty of Medicine and Health Sciences, Université de Sherbrooke, Sherbrooke, QC J1H 5N4, Canada; 4 Department of Medical Biology, CIUSSS of Saguenay-Lac-Saint-Jean, Saguenay, QC G7H 7K9, Canada; 5 Department of Population Medicine, Harvard Pilgrim Health Care Institute, Harvard Medical School, Boston, MA 02215, USA; 6 Diabetes Unit, Massachusetts General Hospital, Boston, MA 02114, USA

**Keywords:** body mass index, childhood adiposity, cord blood leptin, dual-energy X-ray absorptiometry, sex-specific associations, skinfolds

## Abstract

Lower cord blood leptin levels have been associated with lower and higher adiposity in childhood and associations seem to differ according to the child’s age, methods of adiposity assessment and sex. Our aim was to investigate sex-specific associations of cord blood leptinemia with childhood adiposity at birth, 3 and 5 years of age. We measured cord blood leptin using Luminex immunoassays in 520 offspring from the Gen3G cohort. We tested associations between cord blood leptin and body mass index (BMI) z-score, skinfolds thicknesses (SFT), and body composition using dual-energy X-ray absorptiometry, adjusted for confounders. At birth, girls had almost twice as much leptin in cord blood as boys (15.5 [8.9; 25.6] vs. 8.6 [4.9; 15.0] ng/mL; *p* < 0.0001) as well as significantly greater adiposity. Lower levels of cord blood leptin were associated with higher sum of SFT (β = −0.05 ± 0.02; *p* = 0.03) and higher BMI z-score (β= −0.22 ± 0.08; *p* = 0.01) in 3-year-old boys only. We did not observe these associations at age 5, or in girls. Our results suggest a sexual dimorphism in the programming of leptin sensitivity and childhood adiposity, but further observational and functional studies are needed to better understand the role of leptin in early life.

## 1. Introduction

In 2020, 39 million children under 5 years of age were overweight or obese across the world, according to the World Health Organization (WHO) [1]. Given that childhood obesity is a strong predictor of adult obesity [2], it is crucial to identify children at risk of developing obesity as early as possible and to understand the biologic determinants of early life weight regulation.

Leptin is a hormone secreted by adipocytes and one of its biological effects is to regulate food intake and increase energy expenditure, in part via its action on arcuate nucleus (ARC) neurons in the hypothalamus [3,4,5]. Indeed, an important physiological function of leptin is to signal to the central nervous system (CNS) a decrease in energy storage when leptin levels decline or are low [5,6]; the CNS then responds accordingly by triggering a positive energy balance which will increase appetite and reduce energy expenditure [6]. In the ARC, leptin activates anorexigenic neurons expressing pro-opiomelanocortin (POMC) and cocaine- and amphetamine-related transcript and inhibits orexigenic neurons expressing neuropeptide Y and agouti-related protein [5,6]. Interestingly, leptin levels are high (hyperleptinemia) in humans with common obesity without affecting appetite and body weight regulation, probably indicating a state of leptin resistance in the CNS [7,8].

The regulation of leptin secretion and action in the perinatal period is of high interest. In animal models, there is an important increase in the level of leptin during the first week of life [9]—the leptin surge—which is critical for the development of neuronal circuits regulating food intake in the hypothalamus [4,10]. Leptin deficiency at birth can also impair the formation of neurons projections that are involved in energy homeostasis from the ARC to the paraventricular nucleus [10]. Although pregnancy is associated with elevated maternal leptin levels in both rodents [5] and humans [6], maternal leptin cannot cross the placenta and therefore does not contribute to cord blood leptin levels [11].

Some human studies have reported that the level of leptin in cord blood was associated with adiposity in children at birth [11,12,13,14] and later in life [12,13,15,16,17,18,19,20], suggesting that cord blood leptin might be a potential predictor of childhood adiposity. However, these studies differed in their conclusions about the direction of the association (lower cord blood leptin being associated with both lower and higher adiposity in childhood), sometimes varying according to the timing of the adiposity measurements during childhood and whether adiposity was measured using simple anthropometry (body mass index [BMI], BMI z-score or skinfold thicknesses [SFT]) [12,15,16,17,19] or medical imaging technologies (magnetic resonance imaging [MRI] or dual-energy X-ray absorptiometry [DXA] which are gold standard for body composition in children) [13,18,20,21]. Moreover, only few studies have investigated sex-specific associations between cord blood leptin and childhood adiposity [15,17,20,21], despite the existence of a clear sexual dysmorphism for circulating leptin and adiposity levels, already detectable at birth [12,22,23,24,25].

To address these gaps, we investigated whether leptin levels in cord blood were associated with adiposity in 3- and 5-year-old children, using anthropometric (BMI z-score and SFT) as well as body composition estimated by DXA and stratified by sex, in a well characterized prospective pre-birth cohort (Gen3G).

## 2. Materials and Methods

### 2.1. Cohort Selection

**Figure 1:**
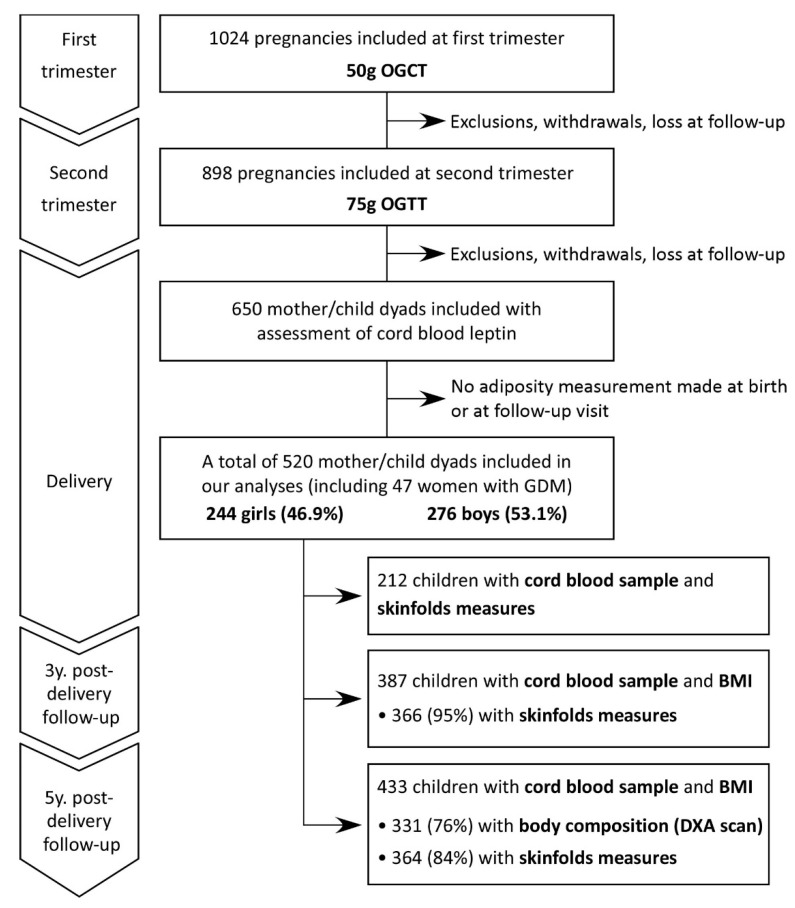
Flow chart of Gen3G participants from enrolment during pregnancy up to the 5 years post-delivery follow-up. BMI, body mass index; DXA, dual-energy X-ray absorptiometry; GDM, gestational diabetes mellitus; OGCT, oral glucose challenge test; OGTT, oral glucose tolerance test.

Our study population included selected mothers and children from the Genetics of Glucose regulation in Gestation and Growth (Gen3G) study. In this prospective observational cohort, pregnant women aged ≥18 years old were invited to participate during the first trimester of pregnancy at the Centre hospitalier universitaire de Sherbrooke (CHUS) in the province of Québec, Canada. As presented in the flow chart (Figure 1), we included a total of 1024 pregnancies between 2010 and 2013. As previously described, exclusion criteria included non-singleton pregnancies, women with pre-pregnancy diabetes or with diabetes diagnosed in the first trimester of pregnancy following the 50g-oral glucose challenge test (OGCT), and women having other conditions or taking medication that could influence glucose regulation [26]. The CHUS ethics committee approved the project, and all women provided a written informed consent, in accordance with the Declaration of Helsinki.

Briefly, at recruitment, we collected data on maternal age, gravidity (primigravid or not), parity (primipara vs. multipara) and smoking habits during pregnancy (yes/no). The research staff measured the height and weight of the participants using standardized protocols and calculated maternal BMI in kg/m^2^. We defined maternal obesity status based on WHO categories (obesity: BMI ≥ 30 kg/m^2^). The participants came back at the research center between the 24th and 28th weeks of gestation for an 75g-oral glucose tolerance test (OGTT). Blood samples were collected at fasting, 1 h and 2 h after glucose ingestion. Women were diagnosed with gestational diabetes mellitus (GDM) according to the International Association of Diabetes and Pregnancy Study Groups (IADPSG) criteria. Hypertensive disorders of pregnancy (HDP) were diagnosed based on national clinical guidelines as reported previously [27]. Gestational weight gain (GWG) was calculated as the measured weight difference between the last medical record entry before delivery and the first trimester visit (V1).

At delivery, we collected data from the medical records on weight (g) and length (cm) of newborns measured by the clinical obstetric staff, and gestational age at birth. Birthweight for gestational age (BW/GA) z-score was calculated using the Fenton’s chart [28]. Our research staff measured skinfolds thicknesses on the right side of newborns at four different sites (triceps, biceps, subscapular and suprailiac) within the first 72 h of life using standardized protocols [29] with a calibrated caliper, in a sub-sample of newborns [26,30]. We performed measurements in duplicate, or in triplicate if the two first measurements varied by more than 10%.

### 2.2. Exposure–Cord Blood Leptin

We collected cord blood samples within the first minutes after delivery and stored aliquots of plasma at −80 °C. We measured cord blood leptin using a multiplexed particle-based flow cytometric assay (Luminex technology; EMD Millipore, Burlington, MA, USA). Intra- and inter-assay coefficient of variation were <10% and <15%, respectively.

### 2.3. Outcomes–Offspring Adiposity Measurements at 3 and 5 Years of Age

At 3- and 5 years post-delivery follow-up visit, our research staff measured children height (cm) using a calibrated stadiometer (Seca) and weight (kg) using a numeric balance (Rice Lake Weighing Systems; model: 140-10-7N), which we used to calculate BMI (kg/m^2^). We computed BMI z-scores for boys and girls using the World Health Organization (WHO) Anthro software for children under 5 years of age [31], otherwise, we used the WHO Anthro Plus software (Growth reference data for 5–19 years) for children over 5 years [32]. In both follow-up visits, we measured children’s triceps, biceps, subscapular and suprailiac SFT (mm) using a skinfold caliper (AMG Medical) in duplicate, or triplicate following the same method mentioned above. The children’s overall adiposity was estimated using the sum of each mean of the four skinfold thicknesses (sum of SFT).

For the 5 years post-delivery visit, we assessed children body composition using a whole-body DXA scan with a Horizon DXA System (Hologic). As previously described, scans were performed by a trained research assistant to ensure correct positioning, absence of movement, and absence of potential artifacts [33]. Of 331 children with a DXA scan and cord blood leptin, movements were suspected for 63 (19.0%) resulting in asymmetric results between right and left limb. As proposed by the Hologic software when asymmetric results occurred, we duplicated data from the limb that did not move (fat mass and lean mass) and applied this data to the limb with suspected movements. Two research team members reviewed each scan with suspected movements to confirm which limb has moved, and to decide if appropriate to duplicate the other limb (mirror) or if the entire scan should be excluded. After careful review and consultation with investigators team when the two initial staff members could not agree, or were still hesitant, no scan were excluded based on excessive movements. We used Hologic software (version 5.5.3.1) to define body regions as instructed in the manufacturer’s manual. For statistical analysis in this study, we used body composition results from DXA scan data such as the percentage of total body fat (total fat mass [g] divided by child’s total mass [g] multiplied by 100), the percentage of trunk fat (trunk fat mass [g] divided by trunk total mass [g] multiplied by 100) and the fat mass index (FMI; total fat mass [kg] divided by child’s height squared [m^2^]).

### 2.4. Statistical Analysis

As presented in the flow chart (Figure 1), for this current study, we included participants for whom we had leptin levels in cord blood and at least one adiposity measurement at any of the time points (Birth: birthweight or SFT; 3 years: BMI z-score or SFT; 5 years: BMI z-score, SFT or DXA). A total of 520 mother-child dyads were included in these analyses. For each variable, we tested the normality of the distribution with the Shapiro–Wilk test and by visualizing histograms. We presented the descriptive characteristics of our population with the number of participants (%) for categorical variables, mean ± standard deviation (SD) for quantitative variables with a normal distribution, or with the median [Q1; Q3] for non-normally distributed variables. We log-transformed non-normally distributed variables to obtain a normal distribution before including in analyses. In the descriptive characteristics table, we compared children by sex (girls vs. boys) using Student’s *t*-test for continuous variables and using equal proportions test (with chi-square) for dichotomous variables. We performed linear regressions to assess associations between cord blood leptin and adiposity measures at birth (birthweight and SFT), 3 (BMI z-score and SFT) and 5 years of age (BMI z-score, SFT and DXA % total body fat, % trunk fat, and FMI). Model 1 was adjusted for sex and child age at the time of adiposity measurements (gestational age in weeks for birth, child age in months for visits in childhood). Model 2 was further adjusted for maternal age, gravidity (primigravid vs. non-primigravid), smoking during pregnancy (yes/no), child’s ethnicity (European descent vs. non-European descent) and maternal BMI at first trimester visit. We explored the influence of maternal BMI on associations between cord blood leptin and child adiposity by conducting sensitivity analyses of Model 2 without maternal BMI. For Model 3, we added other potential confounders: Model 2 covariates + GWG, GDM, gestational age at birth and BW/GA z-score (for birth outcomes analyses, only GWG and GDM were added). We considered HDP as an additional covariate to Model 3; the results of the regression analyses remained essentially unchanged, so we decided not to include HDP in the final models. To assess the effect of child’s sex on associations, we included an interaction term (cord blood leptin x sex) in linear regression model and performed stratified analyses (girls vs. boys). We assessed the relationship between cord blood leptin and each adiposity measure obtained at birth, 3 and 5 years of age using Pearson correlations in boys and in girls separately. *p*-values < 0.05 were considered significant (except for the interaction term where we considered *p* <0.10). All analyses were performed using R version 4.1.0 [34].

## 3. Results

Table 1 presents the characteristics of our 520 mother-child dyads who had cord blood leptin levels and at least one adiposity measurement at birth, 3 or 5 years of age. Included women were 28.3 ± 4.3 years of age at recruitment in the first trimester of pregnancy, 33.3% were primigravid (gravidity) and 48.3% were primipara (parity). A total of 47 (9.0%) women were diagnosed with GDM and 49 (9.5%) had reported smoking during pregnancy. The median maternal BMI, measured by our research staff in the first trimester, was 24.1 [21.6; 28.3] kg/m^2^: 106 (20.4%) women were classified in the obesity category. We observed no difference in the characteristics between mothers who had boys and girls. At birth, we evaluated adiposity with skinfolds in 212 newborns and results showed that girls had significantly greater adiposity, shown by higher values of adiposity for subscapular, suprailiac, and sum of SFT than boys (Table 1). As expected, we found a significant difference in the level of cord blood leptin between sexes: girls (15.5 [8.9; 25.6] ng/mL) had almost twice as much leptin as boys (8.6 [4.9; 15.0] ng/mL; *p* < 0.0001) despite slightly lower birthweight. At 3 years (40.2 [38.4; 42.4] months) and 5 years (64.0 [61.6; 66.5] months) follow-up visits, both boys and girls had similar BMI z-scores, but girls had significantly greater adiposity, shown by higher sum of SFT at age 3 and by higher sum of SFT and % fat (total and truncal) estimated by DXA at age 5.

We first tested the univariate correlations between leptin levels in cord blood (log-transformed) and measures of maternal (during pregnancy) and child adiposity from birth to 5 years of age (Table 2). In both boys and girls, we found that maternal BMI at V1 (r = 0.13) and GWG (r = 0.15 or 0.16) were correlated with cord blood leptin levels. We also found a moderate positive correlation between cord blood leptin levels and birthweight (r = 0.48 or 0.57), BW/GA z-score (r = 0.34 or 0.51) and neonatal SFT (individually [r = 0.30 to 0.44]; sum [r = 0.44 or 0.47]). Higher cord blood leptin levels remained significantly associated with greater adiposity measures at birth after adjusting for multiple confounders in linear regression analyses, including gestational age at birth, child sex, maternal BMI in the first trimester, GWG and GDM (Supplementary Table S1). We observed similar associations in sex-stratified analyses.

At age 3, we did not find correlation between cord blood leptin and adiposity measures in the overall group. However, in boys, we found negative correlations between cord blood leptin and biceps SFT (r = −0.22; *p* = 0.002) and suprailiac SFT (r = −0.18; *p* = 0.01). At age 5, we found that cord blood leptin was positively correlated with adiposity measures based on SFT (r = 0.12 to 0.15; *p* < 0.05) and DXA (r = 0.16 to 0.20; *p* < 0.05) in the overall group (boys and girls combined) (Table 2).

Table 3 shows the associations, for the overall group and sex stratified analyses, between cord blood leptin and adiposity measures at 3 years post-delivery follow-up with results of linear regression adjusted for confounders. We observed potential sex interaction for biceps (interaction *p* = 0.03) and suprailiac (interaction *p* = 0.08) SFT. We found a negative association between cord blood leptin levels and adiposity in boys. In fully adjusted model (Model 3), lower cord blood leptin levels were associated with higher adiposity in boys at age 3, as estimated by SFT measures of biceps (β = −0.09 ± 0.02; *p* = 0.0005), suprailiac (β = −0.10 ± 0.03; *p* = 0.002) and sum of SFT (β = −0.05 ± 0.02; *p* = 0.03). We also observed that lower cord blood leptin was associated with higher BMI z-score in boys (β = −0.22 ± 0.08; *p* = 0.01). We did not find any significant associations in girls. We explored the influence of maternal BMI as covariate (in Model 2) on the associations between cord blood leptin and child adiposity measures: excluding maternal BMI from the models did not substantially change the β estimates in the linear regressions.

Table 4 shows the associations, stratified by sex, between cord blood leptin and adiposity measures at age 5. Our results showed that none of the adiposity outcomes, using anthropometry (individually SFT measures were tested but data is not shown) or DXA, were associated with leptin levels in cord blood in neither boys nor girls.

## 4. Discussion

In this study, we examined the relationship between cord blood leptin levels and adiposity at birth, 3 and 5 years of age and we further investigated these associations in sex-stratified analyses. We found that lower levels of cord blood leptin were associated with lower adiposity at birth in both sexes and were associated with higher adiposity specifically in boys at the age of 3 years. These associations remained similar or were strengthened in models adjusted for many potential confounders, including maternal BMI. We did not observe significant associations between cord blood leptin and adiposity measures at age 5.

Consistent with our results, a recent meta-analysis revealed that cord blood leptin was positively associated with newborn adiposity [11]. Our findings are also in line with two longitudinal cohort studies reporting that cord blood leptin was negatively associated with adiposity measured in children around age 3. First, the Project Viva reported that higher levels of cord blood leptin were associated with lower BMI z-score and sum of subscapular and triceps skinfold thicknesses at age 3 after adjusting for confounding factors (maternal pre-pregnancy BMI, BW/GA z-score, maternal age, level of education, smoking during pregnancy, GWG, paternal BMI, child sex, ethnicity, breast feeding duration, gestational age at birth and child age at adiposity measurements) [16,20], similarly to our fully adjusted model. Second, in line with our findings, the INFAT study found a negative association with fat mass (derived from skinfold equations) at 2 and 3 years of age [12,18]. As in our study, Project Viva assessed body composition using DXA scan and did not find association between cord blood leptin levels and fat mass (kg) or FMI neither at 7 nor 13 years old [20,21]. The INFAT study reported similar results at age 5 in a small subgroup (*n* = 33) and using MRI to assess adipose tissue directly (volumes of abdominal subcutaneous and visceral adipose tissue) [18]. However, and in contrast to findings from Project Viva, INFAT and this current Gen3G study, Simpson et al. showed in the ALSPAC cohort (*n* = 2775) that cord blood leptin was positively associated with DXA fat mass z-score at age 9 after adjusting for multiple confounders, including pre-pregnancy BMI, maternal age, parity, child sex and age (difference in mean per 10 pg/mL: 0.04 standard deviation (SD); [95% confidence interval (CI): 0.00, 0.07]; *p* = 0.023) [13]. However, cord blood leptin became weakly associated after additional adjustment for gestational age at birth, GWG, hypertensive and diabetic disorders during pregnancy (0.03 SD; [95% CI: 0.00, 0.06]; *p* = 0.086) [13]. In summary, our findings in addition to previous studies have shown that the direction of the association between cord blood leptin levels and childhood adiposity varies over time, being positive at birth, negative at 3 years, null at 5 and 7 years and likely positive at 9 years.

Our results are also consistent with the hypothesis of a critical period of leptin sensitivity, followed by a period of resistance to leptin, which was suggested based on observations of repeated adiposity measures in the longitudinal assessment in Project Viva [20]. This concept of a critical window of leptin sensitivity in early life has been demonstrated in animal studies using leptin treatment after birth [10,35] and is consistent with leptin’s role in the development of neuronal circuits regulating food intake in the hypothalamus [6,9,10]. The development of functional connectivity between the nuclei of the hypothalamus occurs in the early postnatal period in rodents [10,36] and after the 34th week of gestation during the third trimester in humans [36,37]. Bouret et al. showed that leptin-deficient ob/ob mice–mice that gain weight rapidly and develop obesity–had a severely diminished innervation in the paraventricular nucleus of the hypothalamus (PVH) by ARC neurons from neonate to adult [10]. However, leptin injection between postnatal day 4 and day 12 in ob/ob mice–to recreate the neonatal leptin surge in rodents [9], which is absent in ob/ob mice–restored the density of ARC neurons projections in the PVH, whereas leptin treatment in adults ob/ob mice did not [10]. Other animal studies have demonstrated that perinatal nutrition induced alterations in hypothalamus via an impaired leptin surge [38]. Studies on maternal undernutrition during gestation and/or lactation (maternal caloric or protein restriction) or postnatal restriction (by increasing the litter size) decreases the level of leptin during the postnatal surge [39,40,41], which has the consequence of reducing the projections of anorexigenic POMC neurons from ARC to PVH in neonates [40,41]. Of note, postnatal leptin surge is not seen in humans as instead leptin levels drop a few days after birth [42]. However, the leptin surge might occur in utero [39] as fetal leptin increases rapidly from the 34th week of pregnancy in human fetuses [43] which corresponds to the period of development of neuronal circuits in the hypothalamus [36,37]. This rise in leptin is hypothesized to be reflecting the rapid increases in fat mass occurring in the fetus during the last weeks of gestation [43].

Therefore, the results from animal studies, previous findings from longitudinal studies and ours might suggest a critical period of leptin sensitivity in early life aimed to program the hypothalamic neural circuits involved in the regulation of food intake and energy expenditure (maybe via rebound or compensatory behaviors) with long-term impacts on fat mass accretion in offspring [11,38,44]. Of note, the strength of the relationship between cord blood leptin and childhood adiposity varies over time. Our and other results have showed that cord blood leptin was strongly and positively associated with all measures of adiposity in newborns. In contrast at age 3, the associations we observed between cord blood with SFT of the biceps, suprailiac and the sum and with BMI z-score in boys were in opposite direction and weaker than the ones observed with adiposity measure at birth. This could be explained by the fact that leptin is a marker of adiposity in cross-sectional assessment (in both boys and girls) at birth, similar to other time points in life, but that if there is an adiposity ‘programming’ phenomenon captured by cord blood leptin levels in humans, this phenomenon is relatively mild and likely attenuated over time. However, since these hypotheses are mainly based on evidence from animal studies with leptin-deficient mice and undernutrition models; we acknowledge major differences between rodents and humans, including development of neural connections not occurring in utero and that postnatal leptin surge is not observed in humans [42]. Therefore, our results must be interpreted with caution.

Based on the very well-known sexual dimorphism of leptin and adiposity levels, we have also tested the associations in both boys and girls separately. In our cohort, we found that newborn girls had significantly higher cord blood leptin levels than boys as previously reported in other studies [12,22,23,24,25]. Furthermore, we observed that girls had significantly more adiposity than boys at all 3 time point measurements (birth, 3 and 5 years). This supports a sexual dimorphism already from the development of fetal adipose tissue since we observed sex differences in adiposity measures at birth, in line with prior studies [45,46]. Indeed, our results suggest that newborn girls had more subcutaneous fat than boys based on skinfold thickness measurements, a method specifically assessing subcutaneous fat mass. Because cord blood leptin derives mainly from fetal adipose tissue [11] and also because leptin levels better correlate with subcutaneous than visceral fat [47], the increased amount of subcutaneous fat in newborn girls could explain why they have higher cord blood leptin levels. Nevertheless, both boys and girls showed similar positive associations between cord blood leptin and adiposity measures at birth. In contrast, the negative associations we observed between cord blood leptin and adiposity at age 3 was present only in boys. We found that a lower level of cord blood leptin was associated with a higher biceps and suprailiac SFT measurements and a higher sum of 4 SFT in boys. This finding was consistent with the Rhea cohort, which found that lower cord blood leptin was associated with higher adiposity among boys at age 4, although they only assessed adiposity with BMI [17]. Opposite to findings from Rhea and our study, results from the MIREC-CD Plus cohort showed that cord blood leptin levels were negatively associated with BMI z-score among 2- to 5-year-old girls–but not in boys–after the model was adjusted for pre-pregnancy BMI, maternal age, paternal BMI, GWG and BW/GA z-score [15]. Although, Project Viva investigated associations between cord blood leptin and childhood adiposity at age 3, 7 and 13, no evidence of sex interactions was found in their analyses, as a result, sex-stratified analyses were not performed [20,21]. Thus, to better understand the impact of our sex-specific association discovery, further studies investigating the relationship between cord blood leptin and childhood adiposity with stratified analyses by sex are needed.

The first strength of our study is the longitudinal design which allowed to assess childhood adiposity with skinfolds thicknesses at birth, 3 and 5 years. Furthermore, we measured body composition with the pediatric gold standard (DXA) in a young cohort of 5-year-old children. Our approach of sex-stratified analyses allowed to add to current literature by showing differences in the associations between cord blood leptin and childhood adiposity in boys versus girls. One limitation of our study is the ethnicity of our population which is composed of 94.4% European descent meaning our results may not be generalized to other population. The observational design only allows us to bring up hypotheses that may explain our findings but prevents us from drawing conclusions regarding causality.

## 5. Conclusions

To conclude, we observed that a lower level of cord blood leptin was associated with lower adiposity at birth and higher adiposity measures in 3-year-old boys but was not associated with adiposity at age 5 even with the assessment of body composition using DXA scan imaging, the gold-standard for pediatric population. Our results also suggest a sexual dimorphism in the programming of leptin sensitivity, more specifically in boys, albeit this seems to be transitory. Further observational studies with sex-stratified analyses and functional studies are needed to better understand the role of leptin in early life.

## Figures and Tables

**Table 1:** Characteristics of 520 mother-child dyads from Gen3G cohort.

	All		Girls (46.9%)		Boys (53.1%)		p *
	Mean ± SD or median [Q1; Q3] or N (%)						
**Maternal characteristics during pregnancy**							
Age, years	520	28.3 ± 4.3	244	28.4 ± 4.5	276	28.3 ± 4.1	0.62
Gravidity, primigravid	520	173 (33.3%)	244	79 (32.4%)	276	94 (34.1%)	0.75 ᵇ
Parity, primipara	520	251 (48.3%)	244	115 (47.1%)	276	136 (49.3%)	0.69 ᵇ
GDM cases	520	47 (9.0%)	244	24 (9.8%)	276	23 (8.3%)	0.66 ᵇ
Smoked during pregnancy	514	49 (9.5%)	242	22 (9.1%)	272	27 (9.9%)	0.86 ᵇ
Hypertensive Disorders of Pregnancy	516	38 (7.4%)	244	20 (8.2%)	272	18 (6.6%)	0.60 ᵇ
BMI at V1, kg/m^2^	520	24.1 [21.6; 28.3]	244	24.0 [21.8; 27.5]	276	24.1 [21.6; 29.3]	0.28 ᵃ
Maternal Obesity at V1	520	106 (20.4%)	244	43 (17.6%)	276	63 (22.8%)	0.17 ᵇ
Gestational weight gain, kg	519	12.0 ± 4.7	244	12.3 ± 5.0	275	11.7 ± 4.4	0.16
**Child characteristics**							
**At birth**							
Gestational age, weeks	520	39.3 ± 1.3	244	39.3 ± 1.3	276	39.3 ± 1.3	0.89
Ethnicity self-reported, European descent	478	451 (94.4%)	223	208 (93.3%)	255	243 (95.3%)	0.45 ᵇ
Birthweight, g	520	3408 ± 468	244	3346 ± 452	276	3463 ± 477	0.004
BW/GA z-score	520	0.07 ± 0.86	244	0.06 ± 0.77	276	0.08 ± 0.93	0.75
Triceps SFT, mm	212	5.0 ± 1.1	97	5.2 ± 1.0	115	4.9 ± 1.1	0.06
Biceps SFT, mm	212	3.8 ± 0.8	97	3.8 ± 0.7	115	3.8 ± 0.9	0.99
Subscapular SFT, mm	212	4.8 ± 1.1	97	5.0 ± 1.1	115	4.7 ± 1.1	0.04
Suprailiac SFT, mm	212	4.2 ± 1.1	97	4.4 ± 1.1	115	4.1 ± 1.1	0.01
Sum of SFT, mm	212	17.8 ± 3.3	97	18.4 ± 3.2	115	17.4 ± 3.4	0.03
Cord blood leptin, ng/mL	520	11.6 [6.0; 19.1]	244	15.5 [8.9; 25.6]	276	8.6 [4.9; 15.0]	<0.0001 ᵃ
**At 3 years post-delivery follow-up**							
Age, months	400	40.2 [38.4; 42.4]	185	40.2 [38.3; 42.1]	215	40.2 [38.5; 42.5]	0.49 ᵃ
BMI, kg/m^2^	387	16.2 ± 1.2	179	16.0 ± 1.1	208	16.3 ± 1.3	0.01
BMI z-score	387	0.53 ± 0.88	179	0.45 ± 0.79	208	0.60 ± 0.95	0.08
Triceps SFT, mm	374	11.2 [9.8; 12.7]	173	11.5 [10.0; 12.8]	201	11.0 [9.5; 12.5]	0.48
Biceps SFT, mm	377	6.5 [5.2; 8.0]	174	7.0 [5.8; 8.7]	203	6.0 [5.0; 7.8]	<0.0001 ᵃ
Subscapular SFT, mm	373	6.0 [5.0; 7.2]	173	6.5 [5.5; 7.8]	200	5.8 [5.0; 7.0]	<0.0001 ᵃ
Suprailiac SFT, mm	366	5.5 [4.5; 7.0]	171	6.2 [5.0; 7.4]	195	5.0 [4.0; 6.0]	<0.0001 ᵃ
Sum of SFT, mm	366	29.5 [25.5; 34.5]	171	31.2 [27.5; 35.5]	195	28.0 [24.8; 32.2]	<0.0001 ᵃ
**At 5 years post-delivery follow-up**							
Age, months	441	64.0 [61.6; 66.5]	208	64.0 [61.5; 66.7]	233	63.9 [61.8; 66.5]	0.84 ᵃ
BMI, kg/m^2^	433	15.6 [14.8; 16.4]	204	15.7 [14.8; 16.4]	229	15.6 [14.8; 16.4]	0.76 ᵃ
BMI z-score	433	0.23 ± 0.97	204	0.23 ± 0.85	229	0.23 ± 1.07	0.95
Triceps SFT, mm	365	11.0 [9.2; 13.0]	172	11.7 [10.0; 13.5]	193	10.2 [8.2; 12.0]	<0.0001 ᵃ
Biceps SFT, mm	366	6.6 [5.0; 8.1]	172	7.2 [6.0; 9.0]	194	5.7 [4.6; 7.0]	<0.0001 ᵃ
Subscapular SFT, mm	364	5.7 [4.9; 6.8]	171	6.3 [5.3; 7.8]	193	5.1 [4.5; 6.0]	<0.0001 ᵃ
Suprailiac SFT, mm	365	6.2 [5.0; 8.2]	172	7.2 [5.8; 9.4]	193	5.7 [4.5; 7.0]	<0.0001 ᵃ
Sum of SFT, mm	364	29.6 [25.1; 35.1]	171	31.8 [28.1; 38.7]	193	26.8 [23.1; 32.1]	<0.0001 ᵃ
DXA trunk fat, %	331	25.5 [22.8; 29.0]	160	27.9 [25.1; 31.2]	171	23.6 [21.5; 26.0]	<0.0001 ᵃ
DXA total fat, %	331	29.9 [27.4; 33.6]	160	32.8 [30.0; 35.3]	171	28.1 [26.1; 29.9]	<0.0001 ᵃ
DXA FMI, kg/m^2^	331	4.6 [4.0; 5.2]	160	4.9 [4.4; 5.5]	171	4.2 [3.9; 4.7]	<0.0001 ᵃ

* Comparisons between groups were performed using the Student’s *t*-Test on normally distributed variables (ᵃ Log-transformed variables) and using tests of equal proportions for dichotomous variables (ᵇ). Gestational weight gain was calculated as the measured weight difference between the last medical record entry before delivery and the first trimester visit (V1). Sum of SFT is the sum of the four skinfolds thicknesses. BMI, body mass index; BW/GA, birthweight for gestational age; DXA, dual-energy X-ray absorptiometry; FMI, fat mass index (fat mass/height-squared); GDM, gestational diabetes mellitus; SD, standard deviation; SFT, skinfold thicknesses; V1: first trimester visit.

**Table 2:** Correlations of Pearson between cord blood leptin (log-transformed) and maternal (during pregnancy) and child adiposity measures (at birth, 3 and 5 years of age).

		All			Girls			Boys		
		*n*	r	*p*	*n*	r	*p*	*n*	r	*p*
	Maternal BMI at V1 (log)	520	0.11	0.01	244	0.13	0.05	276	0.13	0.03
	GWG	519	0.17	0.0001	244	0.15	0.02	275	0.16	0.008
At birth	Birthweight	520	0.45	<0.0001	244	0.57	<0.0001	276	0.48	<0.0001
	BW/GA z−score	520	0.38	<0.0001	244	0.51	<0.0001	276	0.34	<0.0001
	Triceps SFT	212	0.38	<0.0001	97	0.41	<0.0001	115	0.33	0.0004
	Biceps SFT	212	0.29	<0.0001	97	0.34	0.001	115	0.30	0.001
	Subscapular SFT	212	0.46	<0.0001	97	0.44	<0.0001	115	0.44	<0.0001
	Suprailiac SFT	212	0.38	<0.0001	97	0.35	0.0004	115	0.34	0.0002
	Sum of SFT	212	0.47	<0.0001	97	0.47	<0.0001	115	0.44	<0.0001
At 3 years	BMI (log)	387	−0.06	0.27	179	0.00	0.96	208	−0.03	0.67
	BMI z−score	387	−0.05	0.38	179	0.00	0.99	208	−0.03	0.68
	Triceps SFT	374	−0.01	0.82	173	−0.05	0.52	201	−0.01	0.89
	Biceps SFT (log)	377	−0.04	0.46	174	0.01	0.89	203	−0.22	0.002
	Subscapular SFT (log)	373	0.07	0.20	173	0.05	0.52	200	−0.05	0.48
	Suprailiac SFT (log)	366	0.00	0.97	171	0.00	0.99	195	−0.18	0.01
	Sum of SFT (log)	366	0.00	0.94	171	0.00	0.97	195	−0.12	0.09
At 5 years	BMI (log)	433	0.07	0.16	204	0.14	0.05	229	0.01	0.84
	BMI z−score	433	0.06	0.21	204	0.15	0.04	229	0.01	0.85
	Triceps SFT (log)	365	0.12	0.03	172	0.08	0.29	193	0.00	0.95
	Biceps SFT (log)	366	0.13	0.01	172	0.12	0.11	194	−0.04	0.55
	Subscapular SFT (log)	364	0.14	0.006	171	0.10	0.19	193	−0.02	0.82
	Suprailiac SFT (log)	365	0.12	0.02	172	0.03	0.66	193	0.00	0.95
	Sum of SFT (log)	364	0.15	0.005	171	0.09	0.22	193	0.00	0.96
	DXA % Trunk fat (log)	331	0.16	0.003	160	0.01	0.94	171	0.00	0.96
	DXA % Total fat (log)	331	0.20	0.0003	160	0.04	0.58	171	0.06	0.40
	DXA FMI (log)	331	0.17	0.003	160	0.08	0.30	171	0.03	0.67

GWG was calculated as the measured weight difference between the last medical record entry before delivery and the first trimester visit (V1). Sum of SFT is the sum of the four skinfolds thicknesses. BMI, body mass index; BW/GA, birthweight for gestational age; DXA, dual-energy X-ray absorptiometry; FMI, fat mass index (fat mass/height-squared); GWG, gestational weight gain; SFT, skinfold thicknesses; r, Pearson’s correlation coefficient.

**Table 3:** Sex-stratified associations between cord blood leptin (log-transformed) and adiposity outcomes at 3 years of age.

Cord Blood Leptin (log), pg/mL		Triceps SFT mm		Biceps SFT (log), mm		Subscapular SFT (log), mm		Suprailiac SFT (log), mm		Sum of SFT (log), mm		BMI z-Score ᵃ	
		β ± SE	*p*	β ± SE	*p*	β ± SE	*p*	β ± SE	*p*	β ± SE	*p*	β ± SE	*p*
**All** **[*n* = 387]**		*n* = 374		*n* = 377		*n* = 373		*n* = 366		*n* = 366		*n* = 387	
	M1 *	−0.05 ± 0.16	0.77	−0.04 ± 0.02	0.03	−0.002 ± 0.016	0.89	−0.04 ± 0.02	0.07	−0.02 ± 0.01	0.21	−0.04 ± 0.05	0.38
	M2	−0.07 ± 0.16	0.66	−0.04 ± 0.02	0.03	−0.01 ± 0.02	0.71	−0.04 ± 0.02	0.05	−0.02 ± 0.01	0.15	−0.07 ± 0.05	0.15
	M3	−0.02 ± 0.18	0.93	−0.05 ± 0.02	0.01	−0.01 ± 0.02	0.52	−0.07 ± 0.02	0.01	−0.02 ± 0.02	0.12	−0.19 ± 0.06	0.001
	Interaction sex × leptin		0.94		0.03		0.33		0.08		0.20		0.78
**Girls** **[*n*** = **179] (46.3%)**		*n* = 173		*n* = 174		*n* = 173		*n* = 171		*n* = 171		*n* = 179	
	M1	−0.10 ± 0.23	0.66	0.01 ± 0.03	0.82	0.02 ± 0.02	0.48	0.004 ± 0.034	0.90	0.003 ± 0.020	0.89	−0.001 ± 0.072	0.99
	M2	−0.16 ± 0.23	0.50	0.002 ± 0.030	0.95	0.02 ± 0.02	0.46	−0.01 ± 0.03	0.87	−0.003 ± 0.020	0.89	−0.02 ± 0.07	0.76
	M3	0.15 ± 0.27	0.58	0.01 ± 0.04	0.72	0.01 ± 0.03	0.81	−0.02 ± 0.04	0.60	0.01 ± 0.02	0.76	−0.11 ± 0.09	0.21
**Boys** **[*n*** = **208] (53.7%)**		*n* = 201		*n* = 203		*n* = 200		*n* = 195		*n* = 195		*n* = 208	
	M1	−0.04 ± 0.22	0.87	−0.07 ± 0.02	0.002	−0.01 ± 0.02	0.48	−0.07 ± 0.03	0.01	−0.03 ± 0.02	0.09	−0.03 ± 0.08	0.68
	M2	−0.10 ± 0.22	0.67	−0.07 ± 0.02	0.002	−0.02 ± 0.02	0.32	−0.07 ± 0.03	0.01	−0.03 ± 0.02	0.06	−0.09 ± 0.08	0.24
	M3	−0.16 ± 0.25	0.52	−0.09 ± 0.02	0.0005	−0.03 ± 0.02	0.27	−0.10 ± 0.03	0.002	−0.05 ± 0.02	0.03	−0.22 ± 0.08	0.01

M1: Adjusted for child age; M1 *: Adjusted for child age and sex; M2: Model 1 + adjusted for maternal age, gravidity (primigravid vs. non-primigravid), have smoked during pregnancy (yes/no), child ethnicity (European descent vs. non-European descent) and maternal BMI at first trimester visit; M3: Model 2 + adjusted for gestational weight gain, gestational diabetes, gestational age at birth and birthweight for gestational age z-score. Analyses with girls: Model 2 and Model 3 have one observation less for every outcome. Analyses with boys: Model 2 have two observations less for skinfold thicknesses measures and one observation less for BMI z-score; Model 3 have three observations less for skinfold thicknesses measures and two observations less for BMI z-score. ᵃ No correction for child age and sex in models with BMI z-score as an outcome, as these variables were already included in the BMI z-score calculation. Gestational weight gain was calculated as the measured weight difference between the last medical record entry before delivery and the first trimester visit (V1). Sum of SFT is the sum of the four skinfolds thicknesses. BMI, body mass index; SE, standard error; SFT, skinfold thicknesses.

**Table 4:** Sex-stratified associations between cord blood leptin (log-transformed) and adiposity outcomes at 5 years of age.

Cord Blood Leptin (log), pg/mL		DXA Trunk Fat (log), %		DXA Total Fat (log), %		DXA FMI (log), kg/m²		Sum of SFT (log), mm		BMI z-Score ᵃ	
		β ± SE	*p*	β ± SE	*p*	β ± SE	*p*	β ± SE	*p*	β ± SE	*p*
**All** **[*n* = 433]**		*n* = 331		*n* = 331		*n* = 331		*n* = 364		*n* = 433	
	M1 *	0.000 ± 0.009	1.00	0.01 ± 0.01	0.33	0.01 ± 0.01	0.31	0.01 ± 0.02	0.45	0.06 ± 0.05	0.21
	M2	0.000 ± 0.009	0.97	0.01 ± 0.01	0.41	0.01 ± 0.01	0.51	0.01 ± 0.02	0.74	0.04 ± 0.05	0.47
	M3	−0.002 ± 0.010	0.88	0.002 ± 0.009	0.86	−0.01 ± 0.01	0.53	−0.01 ± 0.02	0.46	−0.09 ± 0.06	0.12
	Interaction sex × leptin		1.00		0.93		0.58		0.35		0.22
**Girls** **[*n* = 204]** **(47.1%)**		*n* = 160		*n* = 160		*n* = 160		*n* = 171		*n* = 204	
	M1	0.001 ± 0.016	0.96	0.01 ± 0.01	0.57	0.02 ± 0.02	0.29	0.03 ± 0.03	0.23	0.15 ± 0.07	0.04
	M2	−0.003 ± 0.016	0.83	0.001 ± 0.013	0.91	0.01 ± 0.02	0.59	0.02 ± 0.03	0.52	0.09 ± 0.07	0.22
	M3	−0.002 ± 0.019	0.90	−0.004 ± 0.016	0.79	−0.004 ± 0.024	0.87	0.01 ± 0.03	0.66	−0.01 ± 0.09	0.93
**Boys** **[*n* = 229]** **(52.9%)**		*n* = 171		*n* = 171		*n* = 171		*n* = 193		*n* = 229	
	M1	0.000 ± 0.011	0.98	0.01 ± 0.01	0.37	0.01 ± 0.01	0.63	0.000 ± 0.020	0.98	0.01 ± 0.08	0.85
	M2	0.003 ± 0.011	0.75	0.01 ± 0.01	0.30	0.01 ± 0.01	0.55	0.000 ± 0.021	0.98	−0.004 ± 0.082	0.96
	M3	−0.002 ± 0.012	0.86	0.003 ± 0.011	0.75	−0.01 ± 0.02	0.53	−0.03 ± 0.02	0.24	−0.17 ± 0.09	0.06

M1: Adjusted for child age; M1 *: Adjusted for child age and sex; M2: Model 1 + adjusted for maternal age, gravidity (primigravid vs. non-primigravid), have smoked during pregnancy (yes/no), child ethnicity (European descent vs. non-European descent) and maternal BMI at first trimester visit; M3: Model 2 + adjusted for gestational weight gain, gestational diabetes, gestational age at birth and birthweight for gestational age z-score. Analyses for girls: Model 2 and Model 3 have one observation less for every outcome. Analyses for boys: Model 2 have two observations less for DXA and skinfold thicknesses measures and three observations less for BMI z-score; Model 3 have three observations less for DXA and skinfold thicknesses measures and four observations less for BMI z-score. ᵃ No correction for child age and sex in models with BMI z-score as an outcome, as these variables were already included in the BMI z-score calculation. Gestational weight gain was calculated as the measured weight difference between the last medical record entry before delivery and the first trimester visit (V1). Sum of SFT is the sum of the four skinfolds thicknesses. BMI, body mass index; DXA, dual-energy X-ray absorptiometry; FMI, fat mass index; SE, standard error; SFT, skinfold thicknesses.

## Data Availability

The data that support the findings of this study are available upon reasonable request to the Gen3G research team.

## Supplementary Materials

The following supporting information can be downloaded at: https://www.mdpi.com/article/10.3390/life12122060/s1, Table S1: Sex-stratified associations between cord blood leptin (log-transformed) and adiposity outcomes at birth.
