# Supplementary material for: Associations between Cord Blood Leptin Levels and Childhood Adiposity Differ by Sex and Age at Adiposity Assessment
Source: Life (Basel). 2022 Dec 8;12(12):2060. doi: 10.3390/life12122060 (PMC9780853; doi:10.3390/life12122060)
Supplement: Supplementary file 1 [file life-12-02060-s001.zip › life-2026990-supplementary.pdf]

Supplementary Table S1. Sex-stratified associations between cord blood leptin (log-transformed) and adiposity outcomes at birth

Cordblood leptin (log), pg/mL		Birthweight, g		Triceps SFT, mm		Biceps SFT, mm		Subscapular SFT, mm		Suprailiac SFT, mm		Sum of SFT, mm	
		β ± SE	*P*	β ± SE	*P*	β ± SE	*P*	β ± SE	*P*	β ± SE	*P*	β ± SE	*P*
All		n = 520		n = 212		n = 212		n = 212		n = 212		n = 212	
	M1*	224.5 ± 19.1	<0.0001	0.44 ± 0.08	<0.0001	0.29 ± 0.06	<0.0001	0.56 ± 0.08	<0.0001	0.45 ± 0.09	<0.0001	1.74 ± 0.25	<0.0001
	M2	213.6 ± 19.3	<0.0001	0.39 ± 0.10	0.0001	0.33 ± 0.07	<0.0001	0.56 ± 0.09	<0.0001	0.44 ± 0.10	<0.0001	1.73 ± 0.26	<0.0001
	M3	199.2 ± 19.8	<0.0001	0.41 ± 0.10	0.0001	0.32 ± 0.07	<0.0001	0.53 ± 0.09	<0.0001	0.41 ± 0.10	0.0001	1.68 ± 0.27	<0.0001
	Interaction sex × leptin		0.33		0.38		0.92		0.68		0.69		0.58
Girls		n = 244		n = 97		n = 97		n = 97		n = 97		n = 97	
	M1	244.8 ± 27.4	<0.0001	0.54 ± 0.13	0.0001	0.27 ± 0.09	0.002	0.61 ± 0.13	<0.0001	0.46 ± 0.14	0.002	1.88 ± 0.38	<0.0001
	M2	222.3 ± 27.8	<0.0001	0.43 ± 0.16	0.01	0.29 ± 0.10	0.004	0.51 ± 0.14	0.001	0.34 ± 0.16	0.03	1.57 ± 0.42	0.0004
	M3	203.7 ± 28.5	<0.0001	0.46 ± 0.16	0.01	0.27 ± 0.10	0.01	0.50 ± 0.14	0.001	0.34 ± 0.16	0.04	1.56 ± 0.41	0.0004
Boys		n = 276		n = 115		n = 115		n = 115		n = 115		n = 115	
	M1	209.5 ± 26.6	<0.0001	0.37 ± 0.11	0.001	0.30 ± 0.09	0.001	0.53 ± 0.10	<0.0001	0.44 ± 0.11	0.0002	1.64 ± 0.33	<0.0001
	M2	205.4 ± 27.0	<0.0001	0.32 ± 0.13	0.01	0.35 ± 0.10	0.001	0.56 ± 0.11	<0.0001	0.48 ± 0.13	0.0004	1.71 ± 0.36	<0.0001
	M3	193.6 ± 27.8	<0.0001	0.35 ± 0.14	0.01	0.36 ± 0.11	0.001	0.52 ± 0.12	<0.0001	0.44 ± 0.14	0.002	1.67 ± 0.39	<0.0001

M1: Adjusted for child age (gestational age at birth); M1*: Adjusted for child age (gestational age at birth) and sex; M2: Model 1 + adjusted for maternal age, gravidity (primigravid vs. non-primigravid), have smoked during pregnancy (yes/no), child ethnicity (European descent vs. non-European descent) and maternal BMI at first trimester visit; M3: Model 2 + adjusted for GWG and gestational diabetes. Analyses for girls: M2 and M3 have 22 observations less for birthweight and 21 observations less for SFT measures. Analyses for boys: M2 have 24 observations less for birthweight and 21 observations less for SFT measures; M3 have 25 observations less for birthweight and 22 observations less for SFT measures. GWG was calculated as the measured weight difference between the last medical record entry before delivery and the first trimester visit (V1). Sum of SFT is the sum of the four skinfolds thicknesses. GWG, gestational weight gain; SE, standard error; SFT, skinfold thicknesses.
